# Effects of miR-146a on the osteogenesis of adipose-derived mesenchymal stem cells and bone regeneration

**DOI:** 10.1038/srep42840

**Published:** 2017-02-16

**Authors:** Qing Xie, Wei Wei, Jing Ruan, Yi Ding, Ai Zhuang, Xiaoping Bi, Hao Sun, Ping Gu, Zi Wang, Xianqun Fan

**Affiliations:** 1 Department of Ophthalmology, Ninth People’s Hospital, Shanghai Jiao Tong University School of Medicine, Shanghai, China

## Abstract

Increasing evidence has indicated that bone morphogenetic protein 2 (BMP2) coordinates with microRNAs (miRNAs) to form intracellular networks regulating mesenchymal stem cells (MSCs) osteogenesis. This study aimed to identify specific miRNAs in rat adipose-derived mesenchymal stem cells (ADSCs) during BMP2-induced osteogenesis, we selected the most significantly down-regulated miRNA, miR-146a, to systematically investigate its role in regulating osteogenesis and bone regeneration. Overexpressing miR-146a notably repressed ADSC osteogenesis, whereas knocking down miR-146a greatly promoted this process. Drosophila mothers against decapentaplegic protein 4 (SMAD4), an important co-activator in the BMP signaling pathway, was miR-146a’s direct target and miR-146a exerted its repressive effect on SMAD4 through interacting with 3′-untranslated region (3′-UTR) of SMAD4 mRNA. Furthermore, knocking down SMAD4 attenuated the ability of miR-146a inhibitor to promote ADSC osteogenesis. Next, transduced ADSCs were incorporated with poly(sebacoyl diglyceride) (PSeD) porous scaffolds for repairing critical-sized cranial defect, the treatment of miR-146a inhibitor greatly enhanced ADSC-mediated bone regeneration with higher expression levels of SMAD4, Runt-related transcription factor 2 (Runx2) and Osterix in newly formed bone. In summary, our study showed that miR-146a negatively regulates the osteogenesis and bone regeneration from ADSCs both *in vitro* and *in vivo*.

Large bone defects may arise from a variety of circumstances such as trauma, congenital deformities or tumor resection. Successful repair of these bone deficiencies still remains a great challenge to surgeons worldwide. As the gold standard for the treatment of bone defects in the clinic, autologous bone grafts are usually limited by considerable donor site mobility and an insufficient supply of bone tissue. Alternatives, such as allografts or metallic implants, have also been used to repair bone defects; however, all of these techniques suffer from multiple disadvantages^1^,^2^,^3^. Recent studies have demonstrated that genetically modified mesenchymal stem cells (MSCs) based tissue engineering therapy gained more momentum in the regeneration of pathologically damaged bone tissues^4^,^5^. Adipose-derived mesenchymal stem cells (ADSCs) have raised a lot of attentions in bone regeneration owing to their easy accessibility and availability in relatively large quantities^6^. However, some researchers argue that ADSCs suffer from limitation of poor efficiency for osteogenesis compared to bone marrow derived mesenchymal stem cells (BMSCs). In order to improve ADSCs’ therapeutic effects on repairing bone defects, many efforts have been made to augment the efficiency of ADSC osteogenic differentiation, including cytokine induction and genetic modifications, with complex intracellular regulatory networks being involved in these processes and complicated molecular mechanisms still need to be elucidated^7^,^8^.

Bone morphogenetic proteins (BMPs) are members of the transformation growth factor β (TGF-β) superfamily and can be divided into 4 subfamilies based on function and sequence^9^. The functions of the BMPs include the induction of chondrogenesis, osteogenesis, and angiogenesis, as well as the synthesis of extracellular matrix proteins^10^. Within this family of proteins, BMP2 has been shown to possess strong osteo-inductive properties that promote the formation of new bone through the initiation, stimulation, and amplification of the bone formation cascade through chemotaxis and by stimulating the proliferation and differentiation of the osteoblastic cell lineage^10^,^11^. BMP2 is produced by many cell types, and it accumulates in the extracellular matrix (ECM). During tissue repair and remodeling processes, BMP2 is released from the ECM, and interacts with nearby cells^12^. BMP2 binds to and activates the cell surface receptor complexes BMPR-I and BMPR-II, then the drosophila mothers against decapentaplegic protein (SMAD) 1/5/8 complex formed in the cytoplasm. Together with the co-activator SMAD4, this complex subsequently translocate into the nucleus to activate important transcriptional factors for osteogenesis such as Runx2, Dlx5 and Osterix^13^,^14^,^15^. Nevertheless, accumulating evidence has indicated that positive, negative, or synergistic effects are observed when the BMPs interacts with the MAPK, Wnt, Hedgehog (Hh), Notch, Akt/mTOR and microRNAs signaling pathways, which orchestrate in the regulation of BMP-induced signaling on bone dynamics^16^. These findings suggest that complicate and intricate intracellular regulatory networks are involved in the regulation of osteoblast phenotype commitment and maturation of osteoprogenitors, and therefore a better understanding of the molecular mechanisms underlying the interaction between BMP signaling and other regulatory components or pathways could provide considerable insights into the regulatory networks involved, and aid in developing efficient approaches to treat bone defects.

MicroRNAs (miRNAs) have been reported to involve in the regulation of a variety of biological processes as a new dimension of gene regulation^17^,^18^. MiRNAs negatively regulate protein expression through binding to specific complementary sequences in the 3′-untranslated region (3′-UTR) of mRNAs^19^. Previous researches have indicated that miRNAs could be induced by regulators that responsible for versatile physiological and pathological processes, and the induced miRNAs in return participate in the regulation of those processes^20^,^21^. Microarray analysis has revealed a series of differentially expressed miRNAs in C2C12 myoblast cell line during the BMP2-induced osteogenic differentiation, and these miRNAs exert negative effects on osteoblast differentiation by regulating the BMP signaling pathway^22^,^23^. These physiological regulations mediated by miRNAs are important for balancing bone formation and resorption processes. However, the diversity of miRNA expression and regulatory function in varying cell types and species also demonstrates the complexity and diversification of miRNA regulation^24^,^25^,^26^,^27^, which still requires in depth exploration. To date, studies concerning the expression profile of differentially expressed miRNAs in response to BMP2 in ADSCs are quite limited. Hence, with the consideration of the important effect of BMP2 on osteogenesis and bone formation, and the prevalence of ADSCs used as seed cell in bone tissue engineering, it is desirable to explore the regulatory roles of the differentially expressed miRNAs in response to BMP2 in ADSCs, and the evolving understanding about this underlying mechanism might provide more effective cell-based therapies for repairing bone defects.

In this study, we screened the miRNAs that were differentially expressed during BMP2-induced ADSC osteogenesis through miRNA microarray analysis. We selected the miRNA that showed with the most significant changes, miR-146a, to analyze its effects on ADSC osteogenic differentiation by lentiviral transduction. We further explored the potential molecular mechanisms related to miR-146a’s effects on ADSC osteogenesis. In addition, we evaluated the effects of miR-146a on bone repair by combining miR-146a-modified ADSCs with a porous poly(sebacoyl diglyceride) (PSeD) scaffold in treating rat critical-sized cranial defect. Our data present new insights into the role of miR-146 in ADSC osteogenic differentiation and bone formation.

## Materials and Methods

### Cell culture

Animal experimental procedures, including isolation of ADSCs, anesthesia, operations and killing of laboratory animals, were performed in strict accordance with the NIH guidelines for the care and use of laboratory animals (NIH Publication No. 85e23 Rev. 1985) and approved by the Animal Experimental Ethic Committee of Ninth People’s Hospital affiliated to Shanghai Jiao Tong University School of Medicine. Primary ADSCs were obtained subcutaneously from the inguinal fat pads of Sprague Dawley rats (female, 6–8 weeks old) using a previously described protocol^28^. Yielded cells were passaged in α-MEM (Invitrogen, Carlsbad, CA, USA) containing 10% fatal bovine serum (FBS, Invitrogen), 100 IU/mL streptomycin and 100 IU/mL penicillin. Flow cytometry was used to characterize ADSCs as previously described^28^. ADSCs were incubated with CD90, CD105, CD166, CD31, CD34, and CD45 (BD Biosciences, San Jose, CA, USA). STAT3 inhibitor WP1066 was purchased from Sigma and ADSCs were treated with WP1066 at a concentration of 5 μM as previously described^29^.

### Microarray assays

Total RNA was harvested from ADSCs cultured in normal medium or ADSCs treated with 200 ng/ml BMP2 (BD Biosciences, San Jose, CA, USA) for 48 hours^7^. The expression of miRNAs was analyzed using Affymetrix GeneChip miRNA 4.0 (Affymetrix Inc, Santa Clara, CA, USA) as previously reported^30^, and both conditions were tested using three parallel replicates.

### Lentiviral construction and transduction

Lentiviral production was performed in Genechem Technology Co., Ltd, China according to a previously reported procedure^31^. The lentiviral vector overexpressing miR-146a was termed Lenti-miR-146a, target sequence containing the stem-loop region was synthesized as shown in Table 1. The lentiviral vector to knockdown miR-146a was named Lenti-miR-146a inhibitor, synthesized sequence to complementarily binding to mature miR-146a was shown in Table 1. The empty vector was used as negative control and termed Lenti-miR-NC. For lentiviral transduction, ADSCs were incubated with an optimal volume of lentivirus supernatant and 5 μg/mL polybrene in Opti-MEM (Invitrogen). After the transduction, ADSCs were added osteogenic medium supplemented with BMP2 (200 ng/ml) for the following experiments.

### siRNA transfection

Small interfering RNA (siRNA) against SMAD4 (si-SMAD4) mRNA were obtained from GenePharma (GenePharma Co., Ltd, China) according to a previous study^32^, sense: 5′ aacagcuaucacuacaaauggccugucuc 3′, antisense: 5′ aagucgauagugauguuuaccccugucuc 3′, negative control siRNA (si-NC) was used as control. Then si-SMAD4 or si-NC was mixed with 5 μl of Lipofectamine 2000 in Opti-MEM (Invitrogen) and the mixture was added to ADSCs cultured in six-well plates. The transfection mixture was changed 8 hours after the transfection^33^.

### Quantitative real-time PCR (qPCR)

Cultured ADSCs were lysed by TRIzol reagent (Invitrogen), and then total RNA isolation and reverse transcription were conducted as previously reported^33^. After reverse transcription, 1 μg of diluted cDNA was used as template, together with Power SYBR Green PCR Master Mix (Applied Biosystems, Foster, CA, USA) and 300 nM gene specific primers (Table 2). qPCR detection was carried out on a QuantStudio^TM^ 6 Flex Real-Time PCR System (Applied Biosystems) and relative expression was analyzed according to Pfaffl method as previously described^34^, in which GAPDH and U6B were utilized as normalization controls.

### Western blot assay

Western blot was conducted according to a previously reported study^35^. Total cell proteins were extracted using protein lysis buffer (Thermo Fisher Scientific Inc., Waltham, MA, USA). Total cell protein concentration was measured using a BCA kit (Pierce, Rockford, IL, USA), then equal amount of proteins were separated in sodium dodecyl sulfate-polyacrylamide gel electrophoresis (SDS-PAGE), separated proteins were then transferred to polyvinylidene fluoride (PVDF) membranes (Merck KGaA, Darmstadt, Germany). The membrane was then incubated with the following primary antibodies: anti-STAT3, anti-phospho-STAT3 (Cell Signaling Technology, Inc. Danvers, MA, USA), anti-OPN, anti-Runx2, anti-Osterix, anti-SMAD4, anti-JMJD3 and anti-β-actin (All from Abcam, Cambridge, MA, USA). Then the membrane was incubated with Dylight^TM^ 800 secondary antibody (Invitrogen), the immunoblots were imaged by LI-COR Odyssey infrared Imaging System (LI-COR, Lincoln, NE, USA).

### ALP, ARS staining and activity assay

Alkaline phosphatase activity and extracellular matrix calcification was examined by ALP staining and Alizarin Red-S staining on days 7 and 14 as previously described^34^. Briefly, cells were fixed and then stained using an Alkaline Phosphatase Staining Kit (CosmoBio Co., Ltd. Japan) or Alizarin Red-S solution (Sigma) according to the manufacturer’s recommendations. The semi-quantitative analyses of ALP and ARS were performed following an established protocol^32^, for ALP measurements, cells were lysed and cell supernatant was collected, then substrate p-nitrophenyl phosphate (p-NPP) (Sigma) was added, the optical density (OD) values were measured using a spectrophotometer (Bio-Rad Labtoratories, Inc. Hercules, CA, USA) at 405 nm. For ARS measurements, the ARS stain was dissolved in cetylpyridinium chloride (10%) for 1 hour, then the solution was collected and absorbance reading was performed at 590 nm. Finally, an absorbance index was calculated by normalizing ALP or ARS levels to the total cell protein content.

### Immunocytochemistry

Immunocytochemistry was performed according to a previously reported study^36^. In brief, transduced ADSCs were washed with phosphate saline (PBS, Invitrogen) and fixed in 4% paraformaldehyde (Sigma), then permeabilized in Triton X-100 (1%, Sigma) for 10 minutes. Specific primary antibodies targeting Runx2 and Osterix (1:200, all from Abcam) were incubated overnight before using species-specific fluorescence-conjugated secondary antibody (1:800, Abcam). Nuclei were stained with DAPI (Sigma) before imaged on a confocal microscope (Leica microsystems, Heidelberg, Germany). Positive cell ratio was evaluated by dividing the number of Runx2- or Osterix-positive ADSCs by the number of DAPI-stained ADSCs, respectively.

### Luciferase assays

Predicted targets of rno-miR-146a were identified using miRanda (www.microrna.org) and TargetScan (www.targetscan.org). The 3′ UTR fragment of SMAD4 (NM_019275) carrying the potential binding site (positions 385–392) or its mutant sequence was synthesized (Table 1) and inserted downstream the luciferase gene of pGL3-control vector (Promega Corporation, Fitchburg, WI, USA), termed wt-SMAD4-3′-UTR and mut-SMAD4-3′-UTR, respectively. Constructed reporter vectors were co-transfected with pRL-TK vector (Promega) and miR-146a overexpression plasmid or its control plasmid using X-tremeGENE transfection reagent (F. Hoffmann-La Roche Ltd, Basel, Switzerland) into 293 T cells. After 48 hours, transfected cells were harvested and measured for luciferase activity using a luciferase reporter assay system (Promega)^37^.

### Surgical procedures

All procedures were performed in strict accordance with the NIH guidelines for the care and use of laboratory animals (NIH Publication No. 85e23 Rev. 1985) and approved by the Animal Experimental Ethic Committee of Ninth People’s Hospital affiliated to Shanghai Jiao Tong University School of Medicine. Twenty-four SD rats (female, 6–8 weeks old) were divided randomly into four groups: (A) PSeD scaffold loaded with miR-146a transduced ADSCs; (B) PSeD scaffold loaded with miR-146a inhibitor transduced ADSCs; (C) PSeD scaffold loaded with miR-NC transduced ADSCs; (D) PSeD scaffold alone as the negative control. The surgery procedure was performed as described in our previous study^28^, briefly, the surgery was carried out under sterile conditions, and animals were anesthetized using pentobarbital sodium (3.5 mg/100 mL) through intraperitoneal injection. Then a sagittal scalp incision was made, followed by the 8 mm critical-sized cranial defect created by a trephine. The scaffold/ADSCs composite was subsequently implanted into the defect before incision was closed using absorbable stitches.

### Micro-computed tomographic evaluation

The samples were harvested 8 weeks after operation and scanned by a micro-computed tomographic imaging system (μCT, GE Explore Locus SP micro-CT, USA) as previously reported^7^. Three-dimensional images were reconstructed to evaluate the repairing effect, besides, three important parameters bone volume fraction (BV/TV), bone mineral density (BMD) and trabecular number (Tb.N) were quantitatively calculated using ABA analysis system.

### Sequential fluorescent labelling

Sequential fluorescent labelling of regenerated bone and mineralized tissue were conducted according to our previous study^8^. Briefly, at 2, 4, and 6 weeks post-operation, the operated SD rats received intraperitoneally sequential fluorescent injections: tetracycline (Sigma, 25 mg/kg), calcein (Sigma, 20 mg/kg) and alizarin red (Sigma, 30 mg/kg).

### Histological and histomorphometric observation

After being examined by micro-CT analyses, the specimens were subjected to dehydration in gradient concentration of alcohol from 80% to 100%, and embedded in PMMA. Longitudinal sections of the samples were cut into approximately 200 μm thick sections using a microtome (Leica) and polished to a thickness of 30 μm for histological observation. The samples were firstly observed for fluorochrome staining using confocal microscope (Leica), and then the sections were stained with Van Gieson’s picro fuchsin for observing regenerated bone tissue as described in our previous study^36^. The images were histomorphometrically evaluated using picture analysis software (Image Pro Plus, Media Cybernetics, USA).

### Immunohistochemistry

Immunohistochemistry was performed according to a previously reported study^38^. Briefly, the samples were first decalcified in 10% ethylene diamine tetraacetic acid (EDTA) and then embedded in paraffin. After the sections were created, a Histostain-SP Kit (Invitrogen) was used according to the manufacturer’s instruction, primary antibodies against SMAD4, Osterix and Runx2 (all from Abcam) were applied to the slides. Positive cell ratios of SMAD4, Runx2 and Osterix were calculated to evaluate the expression levels by dividing SMAD4-, Runx2- and Osterix-positive cell number by total cell number within defect region, respectively.

### Statistical analyses

For miRNA microarray analysis, the RVM t-test was applied to filter the differentially expressed genes for the control and experiment group. After the significant and FDR analyses, we selected the differentially expressed miRNAs according to the p-value threshold (p < 0.05) and fold-change (fold-change > 2). Each experiment was performed at least three repeats, all data were expressed as mean ± standard deviation and statistical significance was analyzed using student’s t-test, p value < 0.05 was deemed to indicate statistical significance.

## Results

### Characterization of rat ADSCs

Rat ADSCs showed high level expression for the MSC markers CD90 (98.9 ± 2.95%), CD105 (98.1 ± 2.86%) and CD166 (99.1 ± 3.67%) whereas the myeloid endothelial cell marker CD31 (1.1 ± 0.43%), haematopoietic lineage marker CD34 (0.6 ± 0.26%) and leukocyte common antigen CD45 (1.67 ± 0.48%) were rarely detected (Supplementary Fig. S1A).

### Microarray analysis

**Figure 1:**
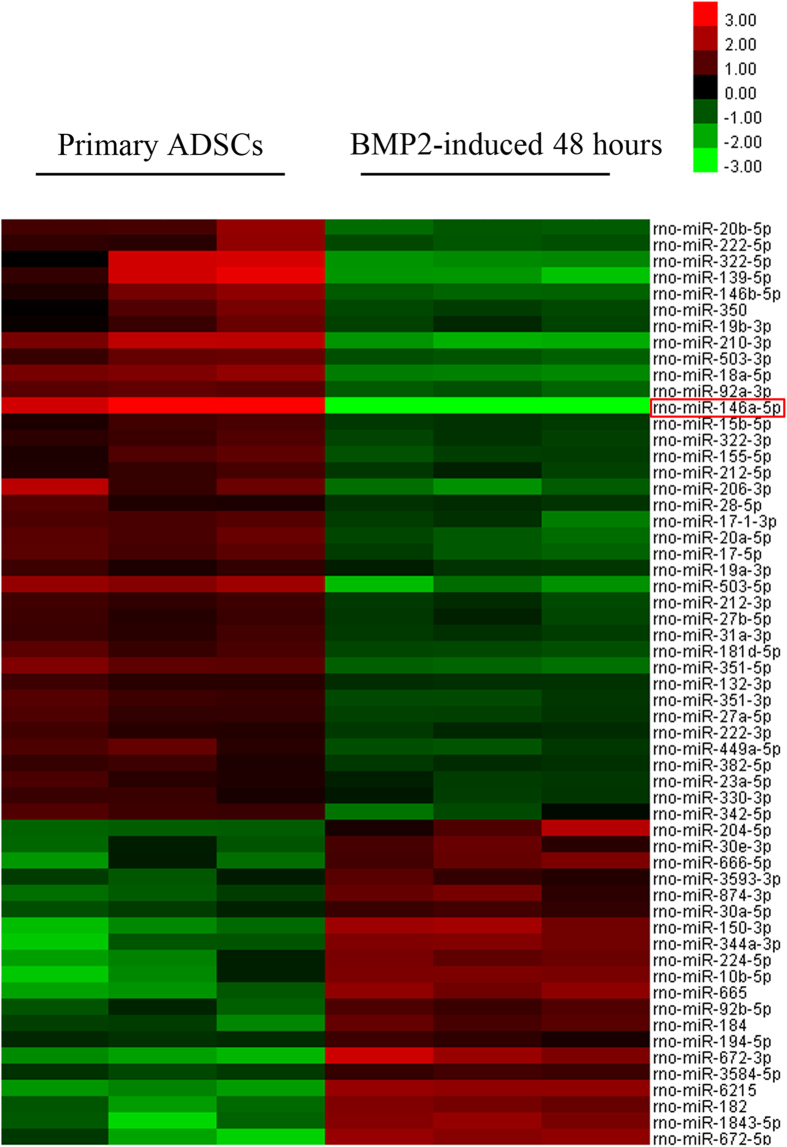
Expression profile of miRNAs in primary ADSCs and ADSCs treated with BMP2 for 48 hours as determined by microarray analysis (n = 3), miR-146a is highlighted by the red box.

To investigate the expression profile of miRNAs in BMP2-treated ADSCs during the osteogenic differentiation process, we analyzed the total RNA extracted from BMP2-treated (48 hours) or non-treated cells on an Affymetrix GeneChip miRNA 4.0 assay. A total of 57 miRNAs were found to be differentially expressed (fold-change > 2, p < 0.05) during the BMP2-induced ADSC osteogenic differentiation, with 37 down-regulated miRNAs and 20 up-regulated. Among these differentially expressed miRNAs, we selected miR-146a because it was the most significantly changed miRNA during BMP2-induced osteogenesis (Fig. 1). To further explore how BMP2 lead to the down-regulation of miR-146a in ADSCs, western blot was performed and showed that the signal transducer and activator of transcription 3 (STAT3) was inactivated after BMP2 treatment in a time dependent manner, and qPCR results showed the expression level of miR-146a decreased gradually following BMP2 treatment (Supplementary Fig. S1B and C). These data suggested that the inactivation of STAT3 might contribute to the down-regulation of miR-146a. To test the role of STAT3 in regulating miR-146a, ADSCs were treated with a specific STAT3 inhibitor (WP1066) and qPCR results showed that intracellular miR-146a level was decreased after the inactivation of STAT3 when compared to the control (Supplementary Fig. S1D). Collectively, our data indicated that BMP2 led to a cohort of differentially expressed miRNAs in ADSCs and miR-146a down-regulation was mediated by the inactivation of STAT3.

### MiR-146a negatively regulates ADSC osteogenesis

To address whether miR-146a regulate the osteoblast differentiation of ADSCs, three lentiviral systems were individually transduced. The emission of green fluorescent protein (GFP) was observed by fluorescence microscopy (Supplementary Fig. S1E) 72 hours after the transduction, the efficiencies of lentiviral transduction of Lenti-miR-146a, Lenti-miR-146a inhibitor and Lenti-miR-NC, which were calculated from the proportion of GFP-positive ADSCs to total cells, were all greater than 80%.

**Figure 2:**
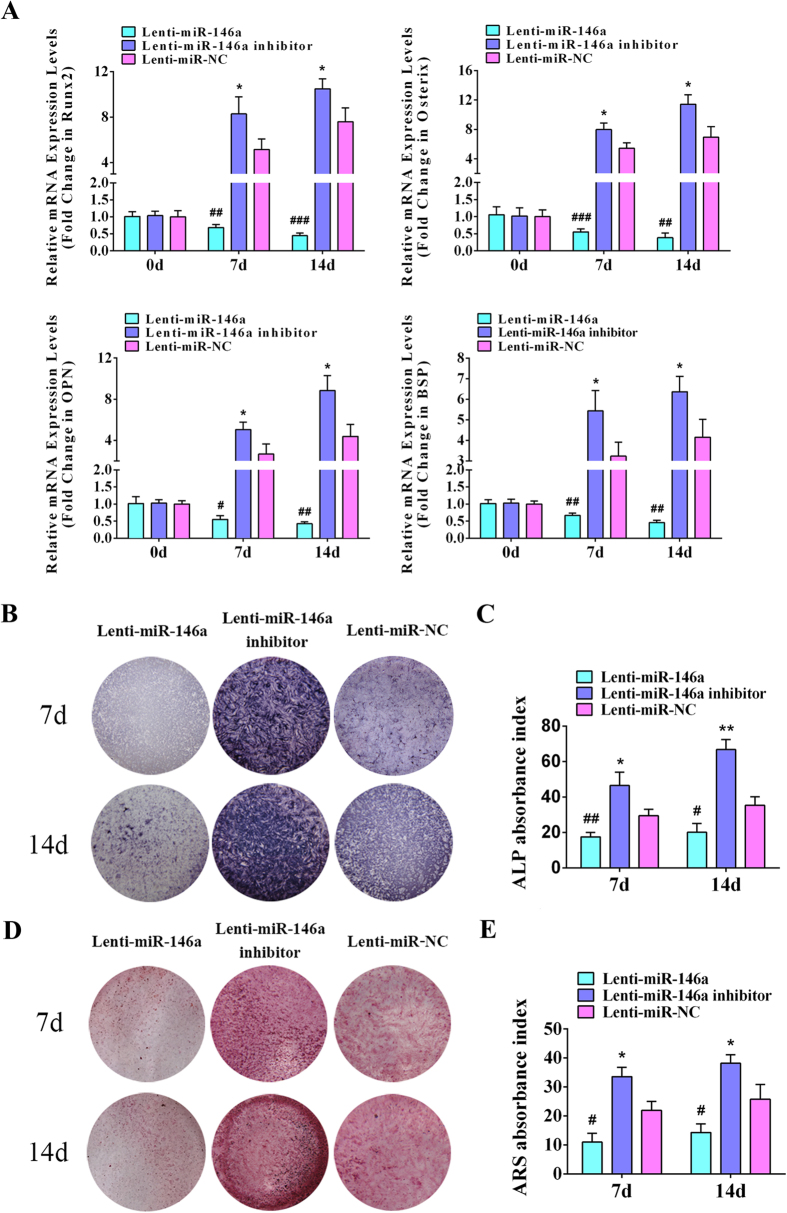
MiR-146a negatively regulates ADSC osteogenesis. (**A**) qPCR analyses of osteogenic differentiation related genes Runx2, Osterix, OPN and BSP in ADSCs transduced with Lenti-miR-146a, Lenti-miR-146a inhibitor and Lenti-miR-NC. (**B**) ALP staining of ADSCs transduced with Lenti-miR-146a, Lenti-miR-146a inhibitor and Lenti-miR-NC on days 7 and 14. (**C**) The ALP activity in ADSCs transduced with Lenti-miR-146a, Lenti-miR-146a inhibitor and Lenti-miR-NC was semi-quantitatively measured on days 7 and 14. (**D**) ARS staining of ADSCs transduced with Lenti-miR-146a, Lenti-miR-146a inhibitor and Lenti-miR-NC on days 7 and 14. (**E**) Semi-quantitative analyses of the extracellular calcium deposition of ADSCs transduced with Lenti-miR-146a, Lenti-miR-146a inhibitor and Lenti-miR-NC on days 7 and 14. ^#^Represents significant differences between the Lenti-miR-146a and Lenti-miR-NC groups; *represents significant differences between the Lenti-miR-146a inhibitor and Lenti-miR-NC groups; ^#^P < 0.05, ^##^P < 0.01, ^###^P < 0.001; *P < 0.05, **P < 0.01, ***P < 0.001.

**Figure 3:**
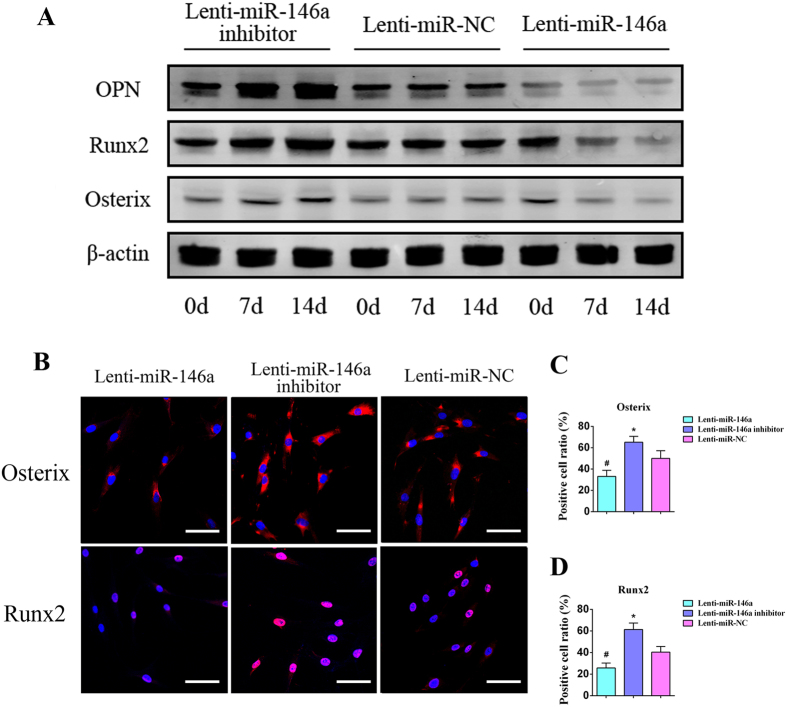
The expression of bone marker proteins is negatively regulated by miR-146a. (**A**) Western blot analyses of OPN, Runx2 and Osterix protein in ADSCs transduced with Lenti-miR-146a, Lenti-miR-146a inhibitor and Lenti-miR-NC. The gels were cropped before displayed in this figure and full-length gels were included in Supplementary Fig. S2. (**B**) Cellular immunochemistry imaged by CLSM shows the expression levels of Osterix and Runx2 in ADSCs transduced with Lenti-miR-146a, Lenti-miR-146a inhibitor and Lenti-miR-NC. Scale bars: 50 μm. Positive cell ratios of Osterix (**C**) and Runx2 (**D**) were determined by dividing the number of immuno-positive cells by the number of nuclei stained with Hoechst. ^#^Represents significant differences between the Lenti-miR-146a and Lenti-miR-NC groups; *represents significant differences between the Lenti-miR-146a inhibitor and Lenti-miR-NC groups. ^#^P < 0.05; *P < 0.05.

qPCR analyses were carried out on days 0, 7 and 14, respectively. A significantly repression on pivotal osteogenic genes such as OPN, Runx2, BSP, Osterix were observed following miR-146a transduction; whereas in ADSCs transduced with miR-146a inhibitor, they were markedly increased on day 7 and continuously elevated on day 14 (Fig. 2A). On days 7 and 14 after gene transduction, ALP staining and quantitative analyses showed that ALP activity in miR-146a overexpressed ADSCs was dramatically attenuated, while greatly enhanced in miR-146a knockdown ADSCs in comparison to the control (Fig. 2B and C). Additionally, experiments were also conducted to detect calcified extracellular matrix, which showed similar tendency to ALP results (Fig. 2D and E). Next, western blot analyses agreed with qPCR results that OPN, Runx2 and Osterix were greatly suppressed in ADSCs transduced with miR-146a at both 7 and 14 days, whereas their expression were significantly increased following the knockdown of miR-146a in ADSCs when compared to the control (Fig. 3A). Cellular immunofluorescence was conducted to detect two pivotal osteogenic transcription factors, Osterix and Runx2, on day 7 after the transduction (Fig. 3B). As shown in Fig. 3C and D, the overexpression of miR-146a significantly repressed the expression of Osterix (33.09 ± 5.76%) and Runx2 (25.69 ± 4.62%) (p < 0.05), while the knockdown of miR-146a increased the expression of Osterix (65.16 ± 5.53%) and Runx2 (61.35 ± 5.98%) (p < 0.05) when compared to the control group [Osterix (49.97 ± 7.27%), Runx2 (40.35 ± 5.27%)]. Collectively, these results indicated that miR-146a negatively regulates the osteogenesis of ADSCs.

### SMAD4 is negatively regulated by miR-146a

**Figure 4:**
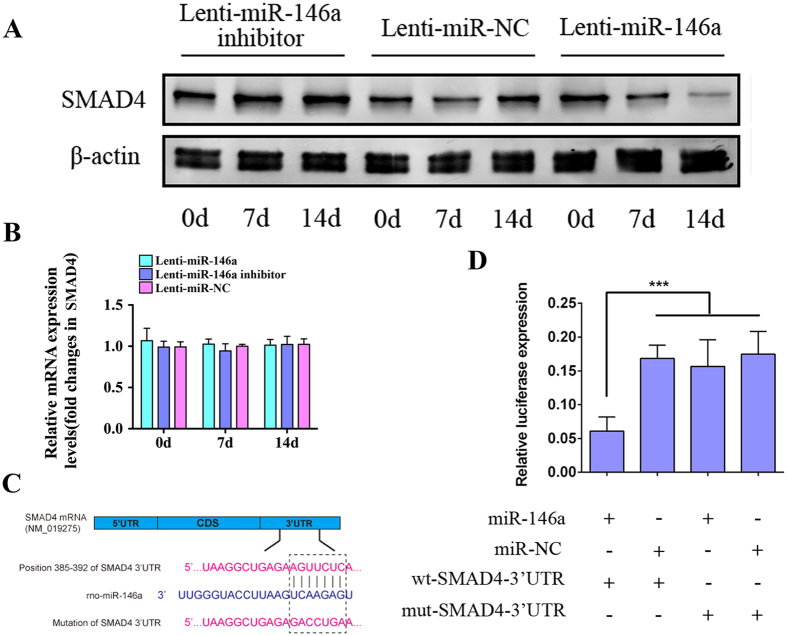
SMAD4 is negatively regulated by miR-146a. (**A**) Western blot analysis showed the expression of SMAD4 following the transduction of Lenti-miR-146a, Lenti-miR-146a inhibitor and Lenti-miR-NC. The gels were cropped before displayed in this figure and full-length gels were included in Supplementary Fig. S3. (**B**) qPCR showed the mRNA expression levels of SMAD4 following the transduction of Lenti-miR-146a, Lenti-miR-146a inhibitor and Lenti-miR-NC. (**C**) Schematic diagram showing the wild type and mutant binding sites of miR-146a located in the SMAD4 3′-UTR. (**D**) The dual luciferase reporter assay showed the relative luciferase expression levels. The firefly luciferase activity data were normalized to Renilla luciferase activity. ***P < 0.001.

Previous report has mentioned that Jimonji-domain containing 3 (JMJD3) is a direct target of miR-146a in human MSCs^39^, to test whether this molecular interaction exists in rat ADSCs, we first screened for the potential target of miR-146a in bioinformatic platforms such as miRanda and TargetScan, the species difference has been taken into consideration that the predicted targets were limited to “Rattus norvegicus”, the results showed us that JMJD3 was not included in the list of potential target of miR-146a. To further test whether miR-146a had regulatory effects on JMJD3 expression in rat ADSCs, we performed qPCR and Western blot analysis, the results showed that neither the mRNA nor the protein expression levels of JMJD3 was affected by the up- or down-regulation of miR-146a (Supplementary Fig. S6). Meanwhile, bioformatic prediction showed that SMAD4 might be one of miR-146a’s potential targets, to investigate whether miR-146a regulates SMAD4 expression, ADSCs were individually assigned to three lentiviral treatments and then subjected to qPCR and western blot analyses. As shown in Fig. 4A, SMAD4 protein expression was gradually decreased in ADSCs overexpressing miR-146a, while the treatment of Lenti-miR-146a inhibitor markedly increased SMAD4 expression when compared to the control. As for the mRNA level of SMAD4, our qPCR results showed that neither overexpressing nor knockdown of miR-146a had significant impacts on SMAD4 mRNA level (Fig. 4B). These data suggested that miR-146a negatively regulates the SMAD4 expression at the post-transcriptional, not the transcriptional level.

To determine the molecular basis of miR-146a’s repression on SMAD4, we again used in silico approaches to identify putative binding site of miR-146a. As shown in Fig. 4C, a conserved binding sequence locates at position 385-392 of the 3′-UTR of SMAD4′s mRNA. To verify this predicted binding site, a dual luciferase reporter system was constructed and co-transfected with the miR-146a overexpression plasmid. As shown in Fig. 4D, luciferase analyses showed that the co-transfection of miR-146a overexpression plasmid (miR-146a) and the wild type 3′-UTR binding site plasmid (wt-SMAD4-3′-UTR) significantly suppressed luciferase expression in comparison to negative control plasmid (miR-NC). However, miR-146a appeared to have no effect on luciferase level when co-transfected with the mutant 3′-UTR binding site (mut-SMAD4-3′-UTR). Taken together, our data suggested that the 385-392 position of SMAD4 3′-UTR is a direct binding site of miR-146a, and miR-146a negatively regulates SMAD4 expression through interacting with 3′-UTR of SMAD4 mRNA.

### SMAD4 knockdown attenuates the effects of the Lenti-miR-146a inhibitor

**Figure 5:**
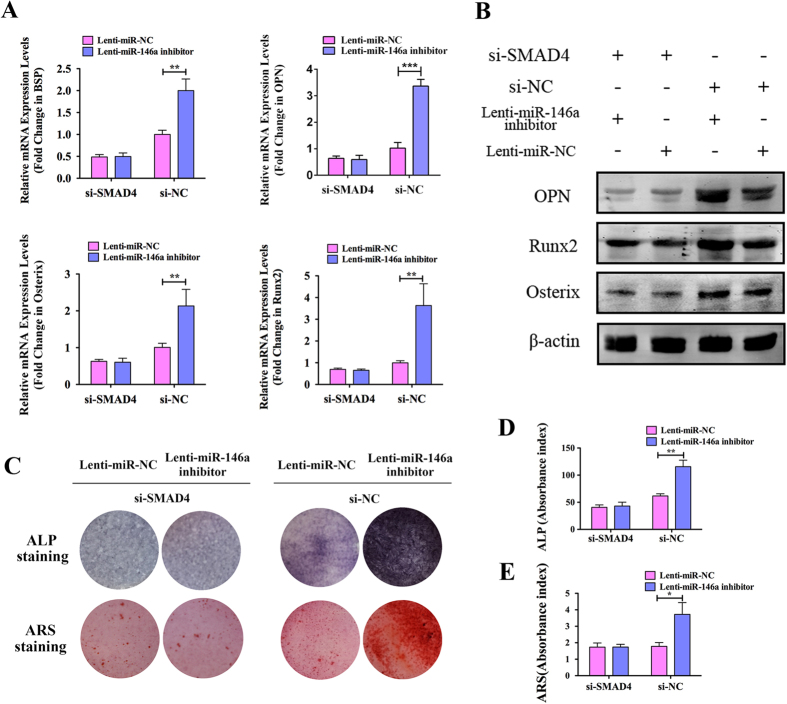
The knockdown of SMAD4 attenuates the effects of the Lenti-miR-146a inhibitor. (**A**) qPCR analyses of the expression of the osteogenic differentiation related genes BSP, OPN, Osterix and Runx2 in ADSCs transduced with Lenti-miR-146a inhibitor or Lenti-miR-NC, with or without SMAD4 knockdown. (**B**) Western blot analyses of Osterix, Runx2 and OPN protein in ADSCs transduced with Lenti-miR-146a inhibitor or Lenti-miR-NC, with or without SMAD4 knockdown. The gels were cropped before displayed in this figure and full-length gels were included in Supplementary Fig. S4. (**C**) ALP and ARS staining in ADSCs transduced with Lenti-miR-146a inhibitor or Lenti-miR-NC, with or without SMAD4 knockdown. (**D**) Semi-quantitative analysis of ALP activity in ADSCs transduced with Lenti-miR-146a inhibitor or Lenti-miR-NC, with or without SMAD4 knockdown. (**E**) Semi-quantitative analysis of extracellular calcium deposition in ADSCs transduced with Lenti-miR-146a inhibitor or Lenti-miR-NC, with or without SMAD4 knockdown. *P < 0.05, **P < 0.01.

To test the relationship between miR-146a and SMAD4, we used a loss-of-function approach in which ADSCs were co-transfected the miR-146a inhibitor, which promotes ADSC osteogenesis, in SMAD4-knockdown ADSCs. qPCR, western blot, ALP and ARS staining analyses were conducted to determine the effects of the miR-146a inhibitor in ADSCs with SMAD4 knocked down. As shown in Fig. 5A, the mRNA expression levels of osteogenic related genes, such as BSP, OPN, Osterix and Runx2 in si-SMAD4 transfected ADSCs were repressed compared to si-NC transfected ADSCs, and these genes could no longer be promoted by Lenti-miR-146a inhibitor. In contrast, the expression of these genes could still be up-regulated by the miR-146a inhibitor in si-NC transfected ADSCs. Furthermore, western blot analyses revealed similar patterns that the protein expression of Osterix, Runx2 and OPN were attenuated in SMAD4-knockdown ADSCs and could not be promoted by the transduction of miR-146a inhibitor (Fig. 5B). In addition, both ALP and ARS staining indicated that ALP activity and extracellular matrix calcium deposition were attenuated in SMAD4-knockdown ADSCs and could no longer be elevated by miR-146a inhibitor, but both were promoted by miR-146a inhibitor in si-NC transfected ADSCs (Fig. 5C). As shown in Fig. 5D and E, semi-quantitative analysis of both ALP and ARS revealed that the knockdown of SMAD4 not only repressed ALP activity and ECM calcification, but also attenuated the effects of miR-146a inhibitor. Taken together, these results indicate that the knockdown of SMAD4 attenuates the effects of miR-146a inhibitor on the osteogenesis of ADSCs.

### MiR-146a regulates bone formation *in vivo*

**Figure 6:**
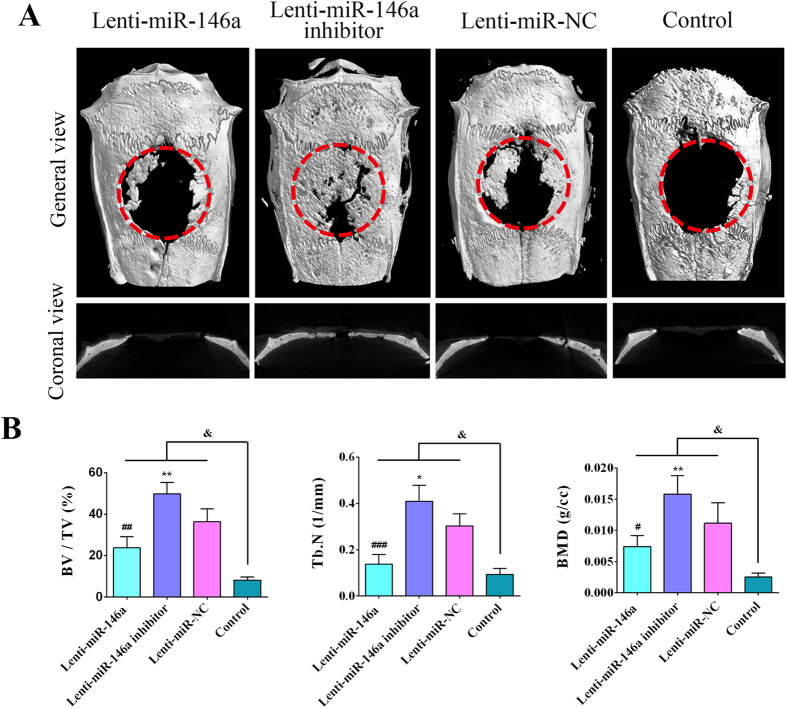
Micro-CT evaluation of the critical-sized calvarial defect repair 8 weeks following the operation. (**A**) 3D reconstruction images depicting the different reparative effects of PSeD scaffold seeded with ADSCs/Lenti-miR-146a, ADSCs/Lenti-miR-146a inhibitor, ADSCs/Lenti-miR-NC and the control group (empty PSeD scaffold). (**B**) Analyses of the percentage of bone volume to total volume (BV/TV), number of trabeculae (Tb.N) and bone mineral density (BMD) in the respective groups. ^#^Represents significant differences between the Lenti-miR-146a and Lenti-miR-NC groups; *represents significant differences between the Lenti-miR-146a inhibitor and Lenti-miR-NC groups; ^&^represents significant differences between the Lenti-miR-146a, Lenti-miR-146a inhibitor, Lenti-miR-NC and the control groups. ^#^P < 0.05, ^##^P < 0.01, ^###^P < 0.001; *P < 0.05, **P < 0.01, ^&^P < 0.05.

To test the effectiveness of miR-146a-modified ADSCs in inducing *in vivo* bone formation, four groups cell-scaffold composites were constructed to repair rat calvarial critical-sized defect by seeding lentiviral-modified ADSCs onto porous poly(sebacoyl diglyceride) (PSeD) scaffolds. Eight weeks post-operation, micro-CT scanning was carried out to observe and analyze the regenerated bone. As Fig. 6A showed, newly formed bone was observed in four groups but highly variable in different group. The amount of regenerated bone in the control group was markedly smaller than the other three groups, there was less regenerated bone in miR-146a treated group, while much more regenerated bone tissue in miR-146a inhibitor treated group in comparison to miR-NC group. Additionally, quantitative analyses (Fig. 6B) showed that significantly higher BV/TV, Tb.N and BMD were detected in miR-146a inhibitor treated group (49.8 ± 5.49%, 0.4094 ± 0.0687, 0.01581 ± 0.00299 g/cc) as compared to the miR-NC group (36.35 ± 6.28%, 0.3029 ± 0.0524, 0.01116 ± 0.0033 g/cc) (p < 0.05); whereas these parameters in miR-146a treated group (23.73 ± 5.47%, 0.1379 ± 0.0409, 0.00743 ± 0.0017 g/cc) were markedly lower (p < 0.05). Parameters in the above three groups were notably higher than the control group (8.23 ± 1.43%, 0.0929 ± 0.0262, 0.00254 ± 0.00059 g/cc) (p < 0.05).

**Figure 7:**
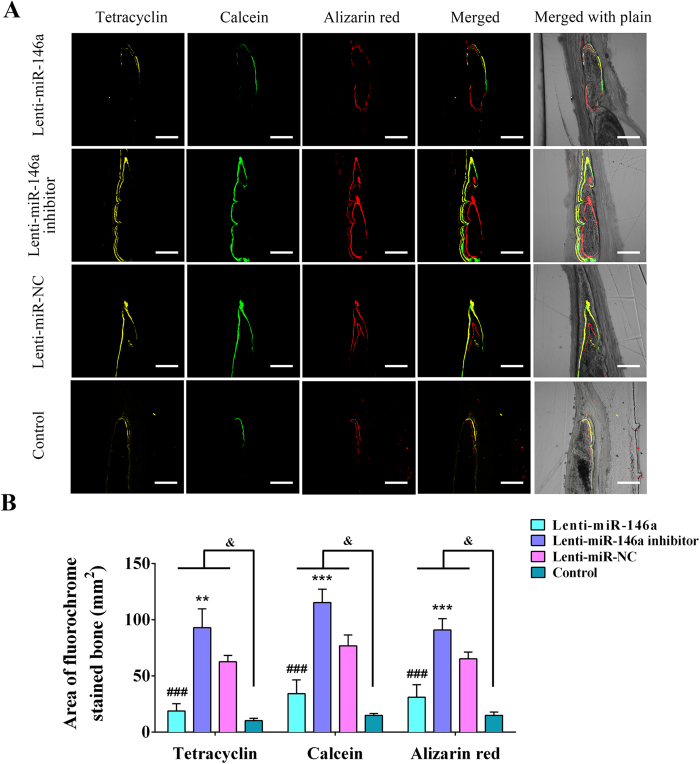
Fluorochrome-labelling analyses of new bone formation and mineralization. (**A**) Line 1 (yellow) shows tetracycline-labelled bone in the respective groups; line 2 (green) shows calcein labelled bone; line 3 (red) shows alizarin red labelled bone; line 4 represents the merged images of the three fluorochromes; and line 5 represents the merged images viewed with a light microscope. (**B**) Analyses of the fluorochrome-labelled new bone area in the respective groups. ^#^Significant differences between the Lenti-miR-146a and Lenti-miR-NC groups; *significant differences between the Lenti-miR-146a inhibitor and Lenti-miR-NC groups; ^&^represents significant differences between the Lenti-miR-146a, Lenti-miR-146a inhibitor, Lenti-miR-NC and control groups. ^##^P < 0.01, ^###^P < 0.001; **P < 0.01, ***P < 0.001; ^&^P < 0.05.

Fluorochrome-labelled histomorphometrical analyses were also processed to evaluate new bone formation and mineralization (Fig. 7A). The different fluorochrome-labelled areas represented regenerated and mineralized bone tissues within weeks 2~4 (tetracycline, yellow), 4~6 (calcein, green) and 6~8 (alizarin red, red). As shown in Fig. 7B, fluorochrome-labelled areas (tetracycline, calcein, alizarin red) in miR-146a treated group (18.85 ± 6.39 mm^2^, 34.22 ± 12.23 mm^2^, 31.1 ± 11.02 mm^2^) were respectively smaller than those observed in miR-NC group (62.58 ± 5.73 mm^2^, 76.83 ± 9.52 mm^2^, 65.18 ± 6.04 mm^2^), while they were markedly larger in miR-146a inhibitor treated group (92.38 ± 16.69 mm^2^, 115.32 ± 11.87 mm^2^, 90.93 ± 9.95 mm^2^) as compared with miR-NC group. These fluorochrome-labelled areas in the control group (10.19 ± 2.07 mm^2^, 14.88 ± 1.71 mm^2^, 14.87 ± 2.99 mm^2^) were markedly smaller when compared to the above three groups (p < 0.05).

Above all, these morphological analyses exhibited that the treatment of miR-146a repressed bone regeneration, whereas the treatment of miR-146a inhibitor significantly enhanced this process, suggesting that miR-146a negatively regulated ADSC-mediated bone formation *in vivo*.

### Immunohistochemical analyses of regenerated bone

**Figure 8:**
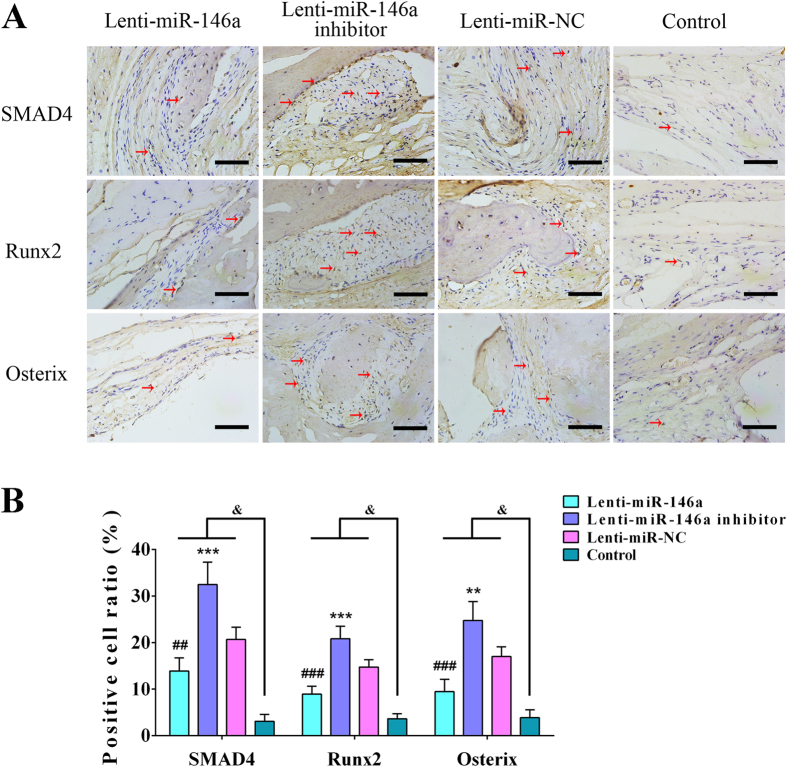
Immunohistochemical analysis of newly formed bone. (**A**) Cells with positively stained cytoplasm were observed as brown nodes, while the cell nuclei were observed as blue nodes. The expression levels of SMAD4, Runx2 and Osterix in Lenti-miR-146a, Lenti-miR-146a inhibitor, Lenti-miR-NC and control groups. Scale bar: 200 μm (**B**) Positive cell ratios of SMAD4, Runx2 and Osterix in newly formed bone were determined by dividing the number of SMAD4-, Runx2- and Osterix-positive cell by the total cell number. ^#^Represents significant differences between the Lenti-miR-146a and Lenti-miR-NC groups; *represents significant differences between the Lenti-miR-146a inhibitor and Lenti-miR-NC groups; ^&^represents significant differences between the Lenti-miR-146a, Lenti-miR-146a inhibitor, Lenti-miR-NC and the control groups. ^##^P < 0.05, ^###^P < 0.01; **P < 0.05, ***P < 0.01, ^&^P < 0.05.

The immunohistochemical analyses were performed to detect SMAD4, Runx2 and Osterix expression in newly formed bone tissue (Fig. 8A). Calculation of positive cell ratios was conducted by dividing SMAD4-, Runx2- and Osterix-positive cell number by total cell number, respectively. As shown in Fig. 8B, the ratios of SMAD4-, Runx2- and Osterix-positive cell in the miR-146a treated group (13.89 ± 2.83%, 8.94 ± 1.64%, 9.46 ± 2.62%) were lower than miR-NC group (20.65 ± 2.62%, 14.74 ± 1.58%, 16.99 ± 2.11%) (p < 0.05), but these ratios in miR-146a inhibitor treated group were much higher in comparison to miR-NC treated group (p < 0.05). The positive ratios of SMAD4, Runx2 and Osterix in the control group (3.09 ± 1.46%, 3.63 ± 1.09%, 3.86 ± 1.71%) was notably lower when compared to the above three groups (p < 0.05). These data suggested that miR-146a regulates bone formation by ADSCs *in vivo* through affecting SMAD4 and its downstream genes including Runx2 and Osterix.

## Discussion

A large number of regulatory and osteoinductive factors have been identified that affect the osteogenic differentiation by activating diverse signaling pathways, and these complex interactive networks have inspired a considerable number of studies^14^,^40^. Among the various osteoinductive factors, bone morphogenetic protein 2 (BMP2) has been proved to be requisite both in skeleton development and bone remodeling process. Recent studies indicate that microRNAs (miRNAs) are important regulators of various biological processes^38^,^41^, in particular, BMP2 induction could significantly change the expressions of multiple miRNAs in diverse cell types, and some of those BMP2-induced miRNAs act as important mediator in the regulation of MSC osteogenesis^7^,^22^. However, previous researches have also indicated the complexity of miRNA regulation, since that both the expression changes and regulatory effects of miRNAs differ across various cell types and species^27^,^33^. Mesenchymal stem cells derived from adipose tissue (ADSCs) are considered as a promising seed cell resource for bone tissue regeneration, therefore, to effectively enhance ADSC osteogenic differentiation and ADSC-mediated bone regeneration, it is important to understand how BMP2 affects the expression of miRNAs in ADSCs and how miRNAs coordinate with intracellular signaling pathways in regulating ADSC osteogenesis. In this study, we treated rat primary ADSCs with BMP2 for 48 hours and then screened for differentially expressed miRNAs during BMP2-induced osteogenesis. Our data revealed that a total of 57 miRNAs were differentially expressed in rat primary ADSCs in response to BMP2 induction, among them 37 miRNAs were down-regulated while 20 were up-regulated. Since study concerning the differentially expressed miRNAs in rat primary ADSCs in response to BMP2 is quite limited, our data might help provide new insights into the altered miRNAs during BMP2-induced osteogenesis. In addition to induce MSC osteogenesis, BMP2 has been demonstrated to elicit multiple effects on a variety of biological processes including cell proliferation and apoptosis. Previous study has indicated that BMP2 induces apoptosis of myeloma cells through the modulation of signal transducer and activator of transcription 3 (STAT3), which is a key factor in mediating immune suppression in tumor microenvironment and responsible for the transcription of several important miRNAs, and the phosphorylated-STAT3 could directly bind to the promoter region of miR-146a and transcriptionally activate its expression^42^,^43^. In our study, both the phosphorylated-STAT3 and miR-146a levels were gradually decreased in BMP2-treated ADSCs, indicating that the inactivation of STAT3 by BMP2 might contribute to the repression on miR-146a. Then, a STAT3 inhibitor further repressed miR-146a expression in ADSCs by the inactivation of STAT3. These data suggested a potential molecular mechanism responsible for the down-regulation of miR-146a in BMP2-treated ADSCs, that BMP2 interacted with STAT3 signaling pathway and subsequently decreased miR-146a expression.

A screen of microRNAs preferentially expressed in osteoblasts identified several miRNAs as important regulators of osteoblast proliferation and/or differentiation, such as miR-34, miR-135 and miR-31^7^,^22^,^24^. In our study, miR-146a was the most significantly down-regulated miRNAs in ADSCs during BMP2-induced osteogenesis. Then, the effects of miR-146a on ADSC osteogenic differentiation was further investigated by overexpression and knockdown experiments. Expression levels of important osteogenic markers were significantly elevated by the knockdown of intracellular miR-146a, together with extracellular matrix calcification enhanced; in contrast, the overexpression of miR-146a led to the opposite effects. Our results suggested that miR-146a negatively regulated the osteogenesis of ADSCs *in vitro*. Except for seed cells, scaffold is another key element for forming tissue-engineering bone. Poly(sebacoyl diglyceride) (PSeD) scaffold has been proved to be bio-compatible and osteo-inductive in our previous study^44^. We seeded the genetically modified ADSCs onto this scaffold, and implanted the composite into the created bone defects in rat crane. Our results showed that the treatment of miR-146a inhibitor markedly improved the repair of calvarial defect, with significantly elevated bone volume fraction (BV/TV), trabecular number (Tb.N) and bone mineral density (BMD) in the regenerated bone tissues; whereas the overexpression of miR-146a attenuated bone regeneration. Additionally, sequential fluorochrome-labelling analyses agreed with the above results. Collectively, our *in vitro* and *in vivo* results suggested that the osteogenic differentiation of ADSCs was negatively regulated by miR-146a, and the knockdown of miR-146a greatly enhanced ADSCs-mediated bone regeneration.

MiRNAs have been proved to exhibit regulatory effects on MSC osteogenesis through regulating key molecules of diverse signaling pathways critical for osteogenic differentiation^45^,^46^. Previous report has made the link between miR-146a and the osteogenesis through negatively regulating JMJD3 in human MSCs^39^. Considering the diversification of miRNA regulation in different species^47^,^48^, we tested whether miR-146a had regulatory effects on JMJD3 in rat ADSCs. Our results showed that neither the mRNA nor the protein expression levels of JMJD3 was affected by miR-146a, suggesting that miR-146a might regulate the osteogenesis of rat ADSCs in a JMJD3-independent way, and the underlying mechanism requires further investigation. SMAD4 has been shown to be a key molecule in the mediation of BMP2-induced osteoblast differentiation^32^,^49^, previous study has revealed that miR-146a is involved in the regulation of chondrocyte apoptosis by targeting SMAD4 in primary rat chondrocytes. Therefore, we hypothesized that this molecular interaction might also exist in rat ADSCs. To verify our hypothesis, we examined the expression of SMAD4 in miR-146a transduced cells. Our data showed that the mRNA levels of SMAD4 were not affected by up- or down-regulation of miR-146a, but the protein levels were negatively regulated by miR-146a, which suggested that SMAD4 expression might be repressed by miR-146a at a post-transcriptional level^31^,^33^. Next, a luciferase activity assay was conducted to show that miR-146a repressed luciferase expression in wt-SMAD4-3′UTR plasmid transfected cells, in addition, mutagenesis of the predicted binding site abolished the inhibitory effects of miR-146a. Our data indicated miR-146a might negatively regulate SMAD4 expression by specifically binding to SMAD4 3′-UTR and interfering with protein translation^50^. However, with the consideration that miRNAs might have multiple targets in gene regulation^25^, to verify whether the repressive function of miR-146a on ADSC osteogenic differentiation was mediated by its repression on SMAD4, we transduced miR-146a inhibitor into SMAD4-knockdown ADSCs, and our results showed that osteogenic markers were no longer elevated by knocking down miR-146a in ADSCs that had also had SMAD4 knocked down^37^. Thus, these results suggested that miR-146a regulated ADSC osteogenic differentiation through affecting SMAD4 *in vitro*. To explore whether this molecular interaction contributed to ADSC-mediated bone regeneration, immunohistochemical staining was performed and showed that SMAD4 expression was also negatively affected by miR-146a in newly formed bone. Moreover, as important downstream genes of SMAD4, Runx2 and Osterix expression showed similar tendency, which is in accordance with both our *in vitro* results and previous reports that the alteration in SMAD4 expression subsequently lead to Runx2 and Osterix changes^49^,^51^.

In order to promote the repair of massive bone defect, the use of bioactive molecules, either somatically injecting or in combination with seed cells and scaffold, has become a major area of interest^52^. Among various factors, BMP2 has been shown to possess strong osteo-inductive properties that induce osteogenic differentiation and new bone formation via diverse molecular mechanisms. Many cell types produce BMP2 during the repair and remodeling processes, in which BMP2 interact with nearby cells to initiate osteogenic process. When BMP2 binds to cell surface receptor, a SMAD 1/5/8 complex together with the co-activator SMAD4 was formed and translocates into the nucleus to activate downstream genes^13^,^53^. Our study demonstrated that knocking down miR-146a significantly promoted SMAD4 expression, this might greatly enhance the transcriptional activity of the SMAD protein complex and subsequently lead to an amplified cascade of expression of downstream osteogenic specific genes, making ADSCs more “sensitive” to commit osteogenesis under the induction of surrounding BMP2, which partly explained the improved reparative effects of miR-146a-knockdown ADSCs. Collectively, our data showed preliminary results that miR-146a could either promote or attenuate ADSC-mediated bone formation, which made miR-146a a novel molecular target for specific therapeutic interventions in curing large bone defects, as well as helping the development of new strategies in preventing pathological ectopic bone formation^54^,^55^,^56^,^57^. Although the development of miRNA-based therapeutic strategies opens up new opportunities for the potential clinical application in the future, however, the nature of miRNA-mediated gene regulation still requires further investigation.

## Conclusion

In summary, our data demonstrated that 57 miRNAs differentially expressed during BMP2-induced osteogenesis in ADSCs, among which miR-146a was the most significantly down-regulated miRNA. The knockdown of miR-146a promoted the osteogenic differentiation of ADSCs by elevating pivotal osteogenic marker genes expression, while the overexpression of miR-146a repressed them. MiR-146a post-transcriptionally regulated SMAD4, a key co-activator in the transduction of BMP2 signaling into the nucleus, through interacting with the 3′-UTR within SMAD4′s mRNA. This regulation on SMAD4 further resulted in alteration of downstream target Runx2 and Osterix expression. The incorporation of PSeD scaffold and miR-146a modified ADSCs holds great potential in developing new strategy for bone defects. Our study provides preliminary results highlighting the prospective application of miR-146a in treating bone related diseases.

## Additional Information

**How to cite this article:** Xie, Q. *et al*. Effects of miR-146a on the osteogenesis of adipose-derived mesenchymal stem cells and bone regeneration. *Sci. Rep.* **7**, 42840; doi: 10.1038/srep42840 (2017).

**Publisher's note:** Springer Nature remains neutral with regard to jurisdictional claims in published maps and institutional affiliations.

## Supplementary Material

Supplementary Data

## Figures and Tables

**Table 1:** Constructed sequences used in this study.

Name	Sequence (5′–3′)
Lenti-miR-146a	accggagagggagtgcctctggatttatcagagatttctcttgtctttacagggctggcaggatctgacctgtgaggaaggaccaggccttacctggagagtctg**tgtgtatcctcagctctgagaactgaattccatgggttatagcaatgtcagacctgtgaagttcagttctttagctgggatagctctatcgtcat**ggacctgaggaacaccgtctaggacaacctctgaaggctttcttattctgccaaggaacttgaagagtggagagagtggggtgaaggatgggactggtggctagc
Lenti-miR-146a inhibitor	aattcaaaaa**tgagaactgaattccatgggtt**ccgg**aacccatggaattcagttctca**tttttg
wt-SMAD4-3′UTR	tctagatatgcgagagagagctccttacatgcaggacactgatgtgttgtctgcagaataaattcactgttgctgactttaaggctgaga**agttctc**aaagttaagtcacctgttacttagtgggcaaagtcattgctgaacgagctgcacgcaagtggcagtccagtcttccaaaagcagcttcactcccttaaagccttttgtcagtctttattcagaatggtgtttgctctaga
mut-SMAD4-3′UTR	tctagatatgcgagagagagctccttacatgcaggacactgatgtgttgtctgcagaataaattcactgttgctgactttaaggctgaga**gacctga**aaagttaagtcacctgttacttagtgggcaaagtcattgctgaacgagctgcacgcaagtggcagtccagtcttccaaaagcagcttcactcccttaaagccttttgtcagtctttattcagaatggtgtttgctctaga

**Table 2:** Primers used for qPCR.

Genes	Accession No.	Forward (5′–3′)	Reverse (5′–3′)	Annealing temperature (°C)	Product size (base pairs)
Runx2	NM_053470.1	tcttcccaaagccagagcg	tgccattcgaggtggtcg	60	154
OPN	NM_012881.2	ttggctttgcagtctcctgcgg	aggcaaggccgaacaggcaaa	60	106
BSP	NM_012587.2	tggagatgcagagggcaaggct	agttggtgctggtgccgttga	60	137
Osterix	NM_152860.1	cggcaaggcttcgcatctg	ggagcagagcagacaggtgaact	60	166
SMAD4	NM_019275	ggctggtcggaaaggattt	ccaggtgagacaacccgctc	60	168
JMJD3	NM_001108829.1	ccctctgtttcctcgtcatc	caagcccgactgatttcttc	60	176
GAPDH	NM_017008.3	aagaaaccctggaccacccagc	tggtattcgagagaagggaggg	60	187
