# Supplementary material for: Effects of miR-146a on the osteogenesis of adipose-derived mesenchymal stem cells and bone regeneration
Source: Sci Rep. 2017 Feb 16;7:42840. doi: 10.1038/srep42840 (PMC5311870; doi:10.1038/srep42840)
Supplement: Supplementary Data [file srep42840-s1.pdf]

Effects of miR-146a on the osteogenesis of adipose-derived mesenchymal stem cells and bone regeneration

Qing Xie[†], Wei Wei[†], Jing Ruan, Yi Ding, Ai Zhuang, Xiaoping Bi, Hao Sun, Ping Gu, Zi Wang*, Xianqun Fan*

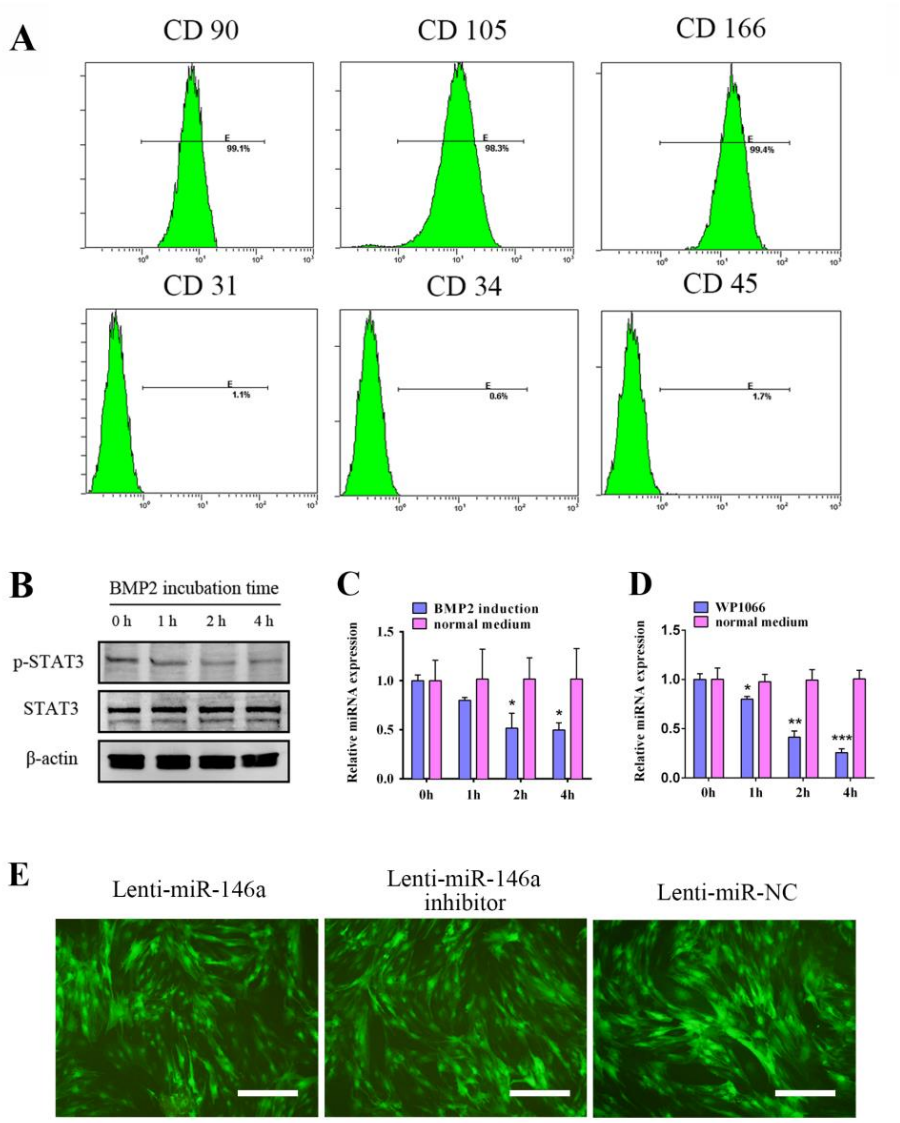

Supplementary Figure S1. (A) Flow cytometry analyses of the surface markers CD90, CD105, CD166, CD31, CD34 and CD45 in ADSCs. (B) Western blot analysis of STAT3 and phosphorylated STAT3 levels in ADSCs treated with BMP2. The gels were cropped before displayed and full-length gels were included in Supplementary Fig. S5 online. (C) qPCR analysis

of intracellular miR-146a levels in ADSCs treated with or without BMP2 at different time points. (D) qPCR results of miR-146a levels in ADSCs treated with or without STAT3 specific inhibitor WP1066. (E) Fluorescence microscope imaging shows GFP-positive ADSCs 72 hours after the transduction of Lenti-miR-146a, Lenti-miR-146a inhibitor and Lenti-miR-NC. Scale bar: 200 μm. * P < 0.05, ** P < 0.05, *** P < 0.01.

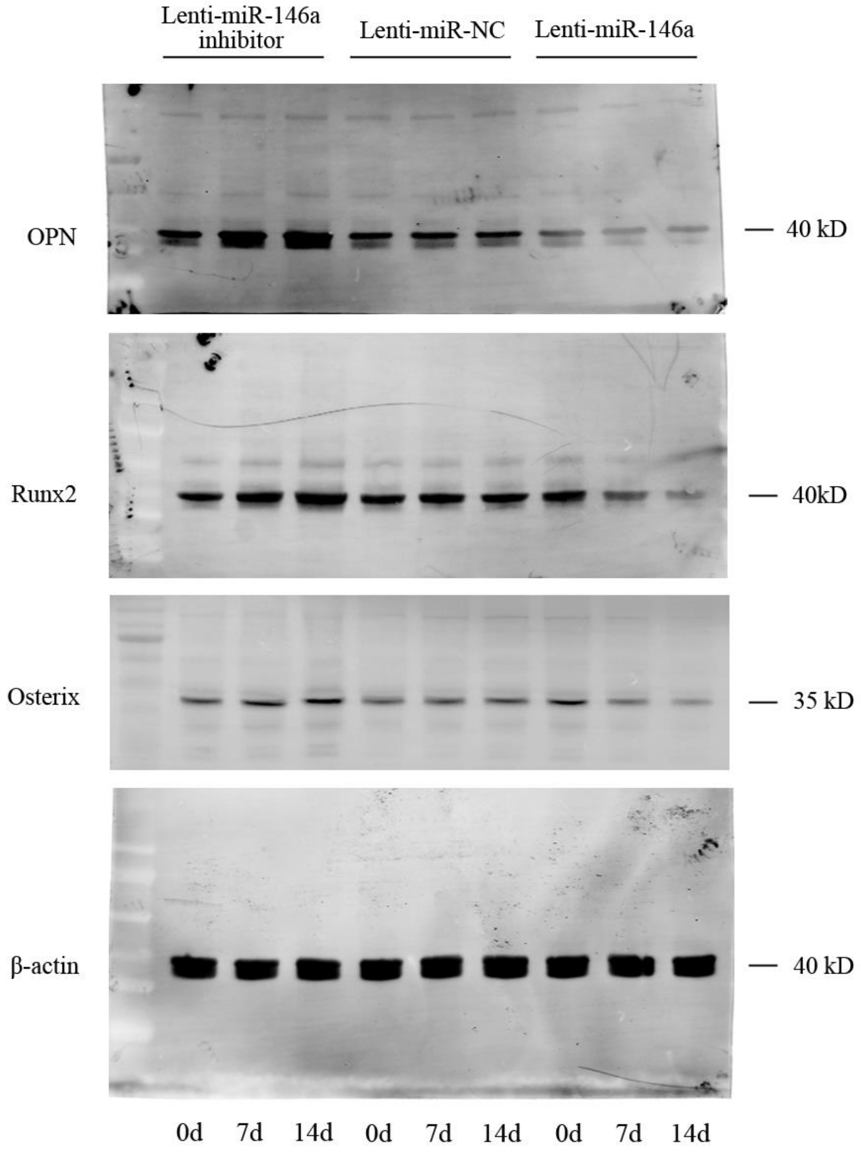

Supplementary Figure S2. Western blot analyses of OPN, Runx2 and Osterix protein in ADSCs transduced with Lenti-miR-146a, Lenti-miR-146a inhibitor and Lenti-miR-NC. Full-length gels were displayed.

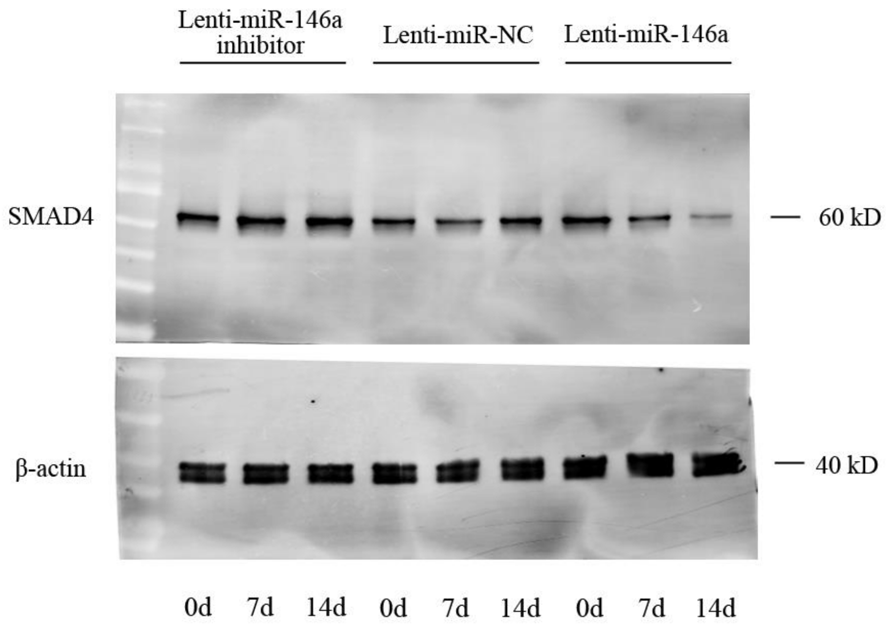

Supplementary Figure S3. Western blot analysis showed the expression of SMAD4 following the transduction of Lenti-miR-146a, Lenti-miR-146a inhibitor and Lenti-miR-NC. Full-length gels were displayed.

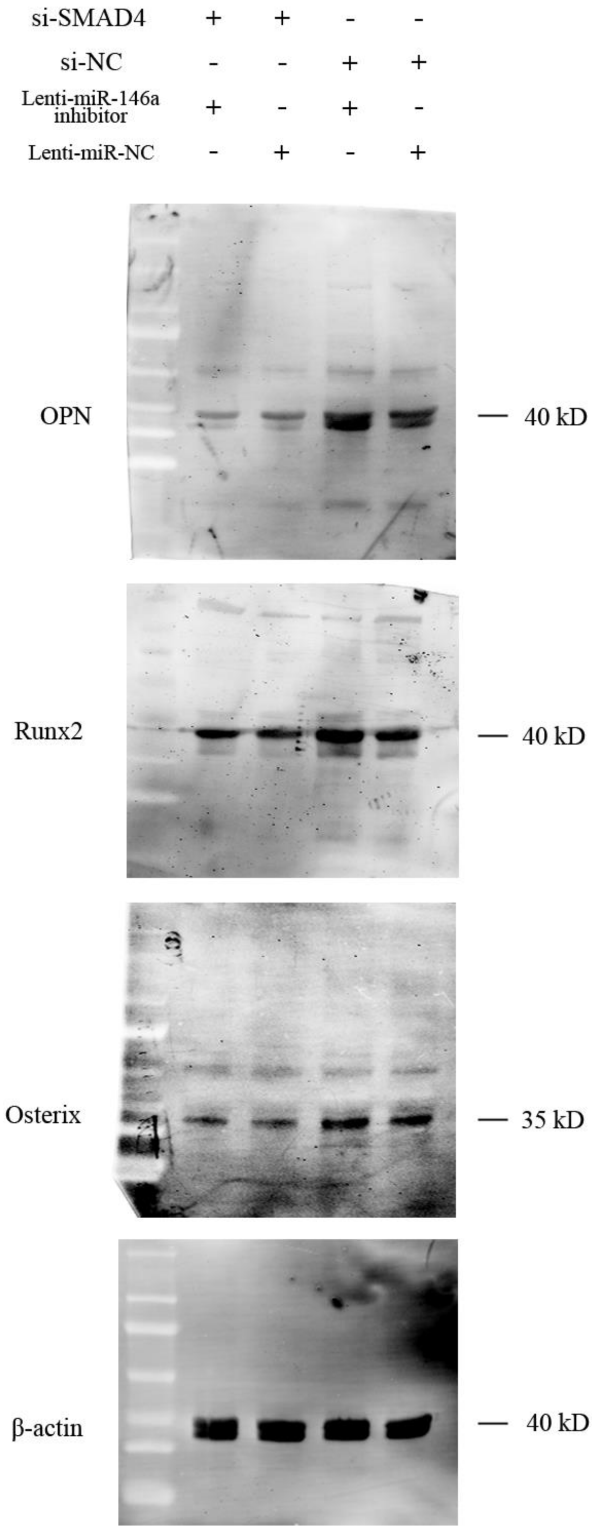

Supplementary Figure S4. Western blot analyses of Osterix, Runx2 and OPN protein in ADSCs transduced with Lenti-miR-146a inhibitor or Lenti-miR-NC, with or without SMAD4 knockdown. Full-length gels were displayed.

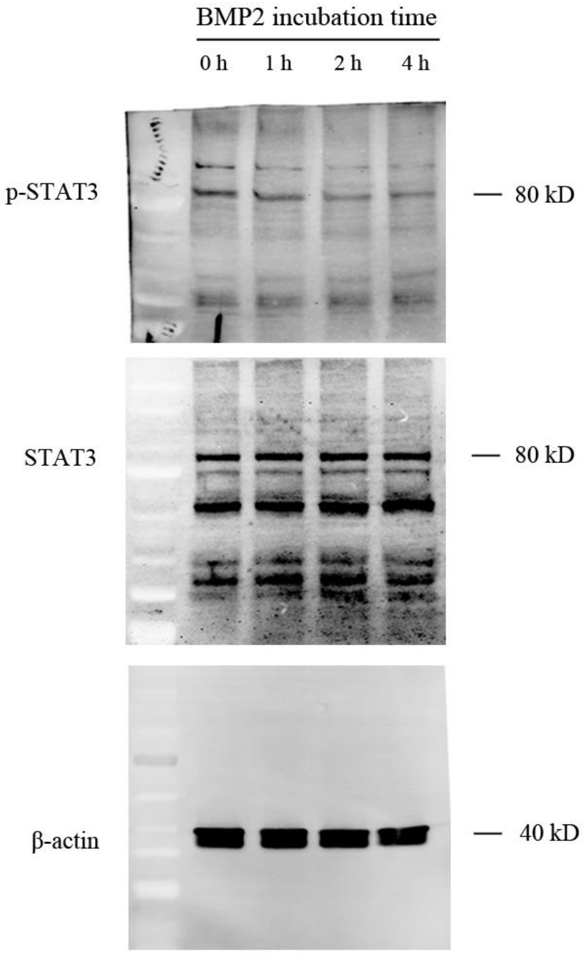

Supplementary Figure S5. Western blot analysis of STAT3 and phosphorylated STAT3 levels in ADSCs treated with BMP2. Full-length gels were displayed.

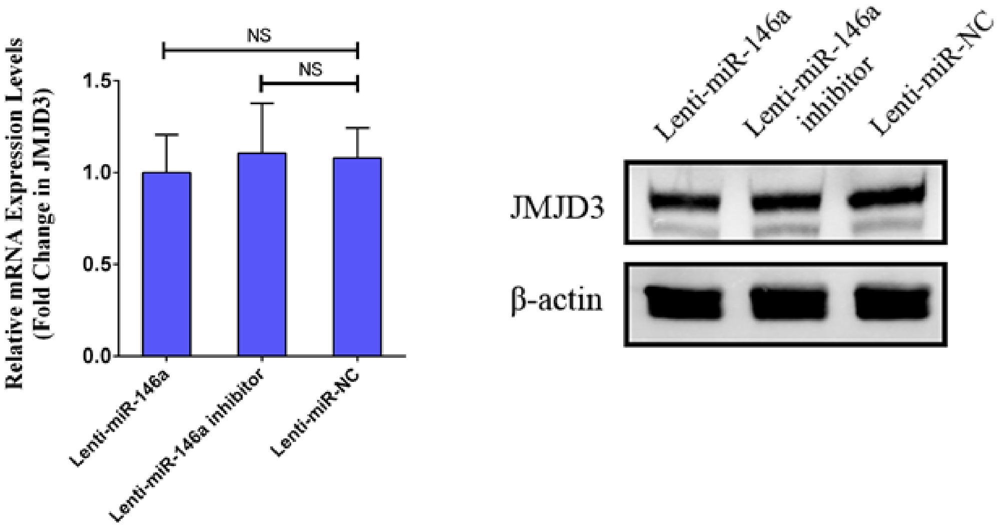

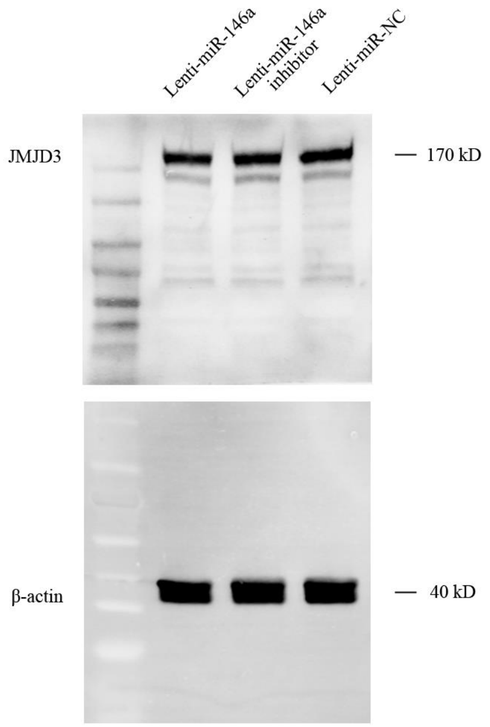

Supplementary Figure S6. qPCR and Western blot analysis of JMJD3 expression after the transduction of Lenti-miR-146a, Lenti-miR-146a inhibitor and Lenti-miR-NC. Full-length gels were displayed, NS indicated no significant difference.
